# A longitudinal exploration of the relationship between obesity, and long term health condition with presenteeism in Australian workplaces, 2006-2018

**DOI:** 10.1371/journal.pone.0238260

**Published:** 2020-08-26

**Authors:** Syed Afroz Keramat, Khorshed Alam, Jeff Gow, Stuart J. H. Biddle

**Affiliations:** 1 Economics Discipline, Social Science School, Khulna University, Khulna, Bangladesh; 2 School of Commerce, University of Southern Queensland, Toowoomba, QLD, Australia; 3 Centre for Health Research, University of Southern Queensland, Toowoomba, QLD, Australia; 4 School of Accounting, Economics, and Finance, University of KwaZulu-Natal, Durban, South Africa; University of Jyvaskyla, FINLAND

## Abstract

**Background:**

Obesity and long term health condition (LTHC) are major public health concerns that have an impact on productivity losses at work. Little is known about the longitudinal association between obesity and LTHC with impaired productivity.

**Objective:**

This study aims to explore the longitudinal association between obesity and LTHC with presenteeism or working while sick.

**Design:**

Longitudinal research design

**Setting:**

Australian workplaces

**Methods:**

This study pooled individual-level data of 111,086 employees collected in wave 6 through wave 18 from the Household, Income and Labour Dynamics in Australia (HILDA) survey. The study used a Generalized Estimating Equation (GEE) model with logistic link function to estimate the association.

**Results:**

The findings suggest that overweight (Odds Ratios [OR]: 1.09, 95% Confidence Interval [CI]: 1.05–1.14), obesity (OR: 1.38, 95% CI: 1.31–1.45), and LTHC (OR: 3.03, 95% CI: 2.90–3.16) are significantly positively associated with presenteeism.

**Conclusions:**

The longitudinal association between obesity and LTHC with presenteeism among Australian employees implies that interventions to improve workers' health and well-being will reduce the risk of presenteeism at work.

## Introduction

The global obesity prevalence has nearly tripled since 1975. In 2016, 13% (over 650 million) of adults aged 18 years and over were obese, worldwide [1]. In 2017–18, nearly 2 in 3 (67%, 12.5 million) Australian adults were either overweight or obese, and 1 in 3 adults was obese [2]. The rising prevalence of overweight and obesity is a serious public health concern in Australia as this trend has high health and financial costs to the economy [3]. In 2015, 8.4% of the disease burden was attributable to overweight and obesity in Australia [2]. Overweight and obesity cost AUD 8.6 billion to the Australian economy in 2011–12 [4].

Excessive weight in workers caused direct (e.g. patient care and medical supplies) and indirect (e.g. lost productivity) cost burdens to employers. The indirect costs of obesity can be grouped into six categories [5] and both absenteeism and presenteeism have contributed highly to indirect costs. Presenteeism is the second main component of measuring workplace productivity and is defined as impaired functioning while being present at work due to the presence of mental or physical health complications [6]. Presenteeism is difficult to identify and measure compared with absenteeism [7]. However, there is evidence that the annual cost of presenteeism is higher than that of absenteeism in the US economy [8]. Like the US, productivity loss through presenteeism is a persistent and ongoing problem in the Australian economy. A landmark study revealed that the estimated cost of presenteeism was AUD 34.1 billion in 2010 and will cost AUD 35.8 billion in 2050 to the Australian economy [7].

It is assumed that obesity negatively impacts workers’ performance as obese people often suffer from comorbidities, including diabetes, cardiovascular diseases, and musculoskeletal disorders. The existing empirical evidence shows that obesity is positively associated with presenteeism [9–13]. Findings from two recent studies conducted in Canada and Belgium suggests that obesity is positively and significantly associated with impaired productivity [10, 11]. Moreover, three studies conducted in the US reported similar findings [9, 12, 13]. One study utilized data of 59,772 adult workers in different US occupations and found that work productivity impairment is significantly higher among obese workers than normal-weight peers [9]. Another study in the US precisely concluded that the rate of presenteeism is 12% higher among obese workers compared with healthy weight counterparts [12]. Similarly, another study of 341 manufacturing employees in the US found that obese workers are less productive than their healthy weight counterparts [13]. The study design of all of these research studies was cross-sectional and based in the US, Canada, or European countries. As a result, a systematic review study suggested conducting a longitudinal study to reconfirm the association between obesity and productivity loss at workplace [5].

No studies have quantified the longitudinal association between workers’ health and impaired productivity. Longitudinal studies can track individual changes over time, and thus can estimate the association more precisely than cross-sectional studies. Additionally, much research has measured presenteeism through a single question and not incorporated important job-related characteristics. To overcome these limitations, the present study aimed to quantify the association between Body Mass Index (BMI) and LTHC with presenteeism using longitudinal data. Three questions will be used to validate the measure of presenteeism. Further, this study will incorporate several health-related, socio-economic, lifestyle, and job-related characteristics as confounders to precisely measure the association. This study may help health policymakers and employers to identify the characteristics of employees associated with a higher rate of presenteeism and make policy interventions to improve workers’ health, thereby improving productivity in the workplace.

## Conceptual framework

**Fig 1:**
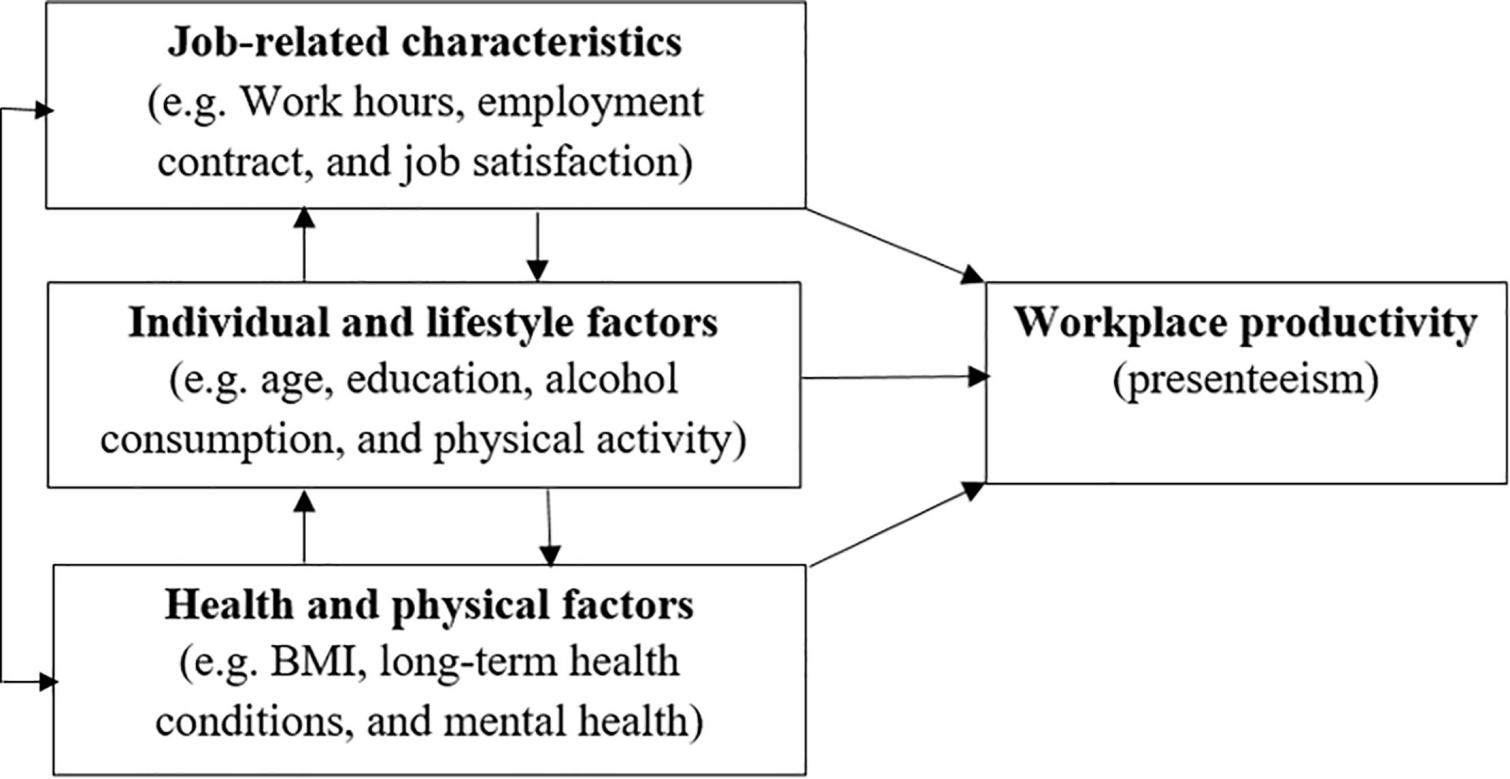
Factors potentially associated with presenteeism. Source: Hafner et al. [14].

To explore the association between obesity and LTHC with presenteeism, this study followed the conceptual framework of Hafner et al. [14]. Fig 1 highlights that factors of workplace productivity are broadly categorized into three groups: job-related factors, individual and lifestyle factors, and health and physical factors. Job-related factors refer to aspects of the work environment, such as work hours, employment contracts, and overall job satisfaction of the workers. Individual and lifestyle factors are related to personal characteristics and behavior, such as age, education, family commitments, alcohol consumption, and physical activity. Health and physical factors include aspects of the health and well-being of the workers, such as weight status, long term health condition, and mental health. The conceptual model posits that job-related characteristics, individual and lifestyle factors, and health and physical factors may have a direct association with workers’ productivity. However, these factors are interrelated dynamically. For example, a worker may develop mental-health problems due to bullying in the workplace. To capture this dynamic effect, Hafner et al. [14] suggested using longitudinal data that can track the same individual over a long period.

## Materials and methods

### Data source and sample selection

The data of the present study were taken from the Household, Income, and Labour Dynamics in Australia (HILDA) survey in Australia. HILDA is a nationally representative household-based panel survey that collects data on three main areas: economic and subjective well-being, labour market dynamics, and family life. More specifically, the survey collects data on a wide range of topics covering family relationships, wealth, income, employment, health, and education [15]. The HILDA survey was commenced in 2001 and since then has been conducted every year. Each year HILDA survey collects data on the lives of over 13,000 Australian adults from more than 7,000 households following a multi-stage sampling approach [16]. The survey collects information from individuals aged 15 years or over in the household through a personal interview by trained interviewers as well as self-completed questionnaires. The details of the survey design have been described previously [15]. The survey is funded by the Australian Government through the Department of Social Services and designed and managed by the Melbourne Institute of Applied Economic and Social Research.

Questions on BMI were included in the HILDA survey from wave 6, and questions on LTHC and presenteeism have been incorporated since wave 1 (see later for details). As a result, the study utilized the most recent thirteen waves (6 to 18) from the HILDA dataset. Given the study’s focus on workplace presenteeism, the analysis was restricted to individuals who are currently employed and aged 15 to 64 years. Further, the study excluded pregnant employees from the subsample analyses to avoid potential bias. Additionally, this study restricted the sample to those with no missing information on the outcome variable (presenteeism) and main exposure variables (obesity and LTHC). After exercising the exclusion criteria, the unbalanced panel consists of 19,087 participants and 111,086 observations for the subsample analysis.

### Outcome variable

The main outcome variable of the present study is presenteeism at work. The variable presenteeism was derived from the Short Form Health Survey (SF-36) questionnaire. The details of the survey can be found elsewhere [17]. Participants were asked three questions through the self-completed questionnaire. More specifically, participants were asked whether they have experienced any of the following three events in the past four weeks due to any physical problems: “cut down the amount of time spent on work or other activities”; “accomplished less than would like”; and “were limited in the kind of work”. The responses were recorded in binary form: yes or no. Using these responses the present study formed a presenteeism variable which is a binary indicator. Presenteeism variable takes the value of 1 if a participant answered “yes” to any of the above three questions, and 0 otherwise.

### Exposure variable

Two health-related characteristics served as the main variables of interest in the present study: obesity and LTHC. The present study used BMI to measure obesity. BMI of the respondents has been derived using the formula weight (in kilograms) divided by square of the height (in meters). BMI has been categorized into four groups following the World Health Organization (WHO) guidelines; underweight (BMI <18.50), normal/healthy weight (BMI 18.50 to <25.00), overweight/pre-obesity (BMI 25.00 to <30.00), and obesity (BMI ≥30) [1]. Underweight is not a concern of the present study. As a result, this study forms a new category, BMI <25, by merging underweight and healthy weight categories following previous studies [18, 19].

The HILDA survey collects data on an individual’s LTHC following the guidelines of the International Classification of Functioning, Disability, and Health (ICF) under the WHO framework [20]. Participants were presented a show-card that listed examples of long term health condition, impairments, or disabilities and asked if they have any of these conditions which restrict them in their daily activities that had lasted or were likely to last six months or more. Responses were taken in binary form, either yes or no. Respondents who answered ‘yes’ were considered as a worker with LTHC, and 0 otherwise.

### Other covariates

This study selected covariates following previous studies on presenteeism at work [10, 11, 21–24]. Socio-demographic covariates included are age (15–35, 36–55, and 56–64 years), gender (male and female), civil status (partnered and non-cohabitating), education (year 12 or below, professional qualification, and university qualification), ethnicity (not of indigenous origin, and Aboriginal or Torres Strait Islander [ATSI]), remoteness (major cities, regional, and remote or very remote), and equivalized household income. Household income variable was categorized into quintiles: quintile 1 (bottom quintile) though 5 (top quintile). In addition to the socio-demographic controls, this study included lifestyle factors and job-related characteristics. Lifestyle factors included smoking status (non-smoker and current smoker), alcohol consumption (non-drinker and current drinker), and physical activity (inactive, some activity, and regular activity). The HILDA survey collects data on an individual’s physical activity by asking how often they participate in physical activity. Responses were taken in 6 forms: not at all, less than once a week, 1 to 2 times a week, 3 times a week, more than 3 times a week, and every day. Respondents who answered ‘not at all’ were classified as inactive, less than once a week, 1 to 2 times a week, and 3 times a week were classified as some activity; and more than 3 times a week and every day were classified as a regular activity.

The present study included the following employment controls: hours worked per week (<35, 35–40, and >40 hours/week), employment contract (permanent, casual, and fixed-term), occupation (8 categories), industry (13 categories), supervisory responsibilities (yes or no), member of employee association (yes or no), provision of paid sick leave (yes or no), and overall job satisfaction (from 0 = worst to 10 = best).

### Estimation strategy

The authors constructed an unbalanced longitudinal data set by linking individual’s records who participated in wave 6 through wave 18 of the HILDA survey. To summarise the characteristics of the cohort, the present study used descriptive statistics in the form of frequency (n) and percentage (%) along with 95% confidence intervals (CI) or mean with standard deviation (SD). Further, this study calculated the frequencies of presenteeism among the study participants by BMI categories, LTHC, and other covariates. Chi-square tests or t-test have been employed to assess the bivariate relationship between presenteeism, obesity, LTHC, and other covariates. This study included covariates in the multivariate analysis if a covariate is significant at p-value equals to 0.05 in the bivariate analysis.

Given the discrete nature of the dependent variable, presenteeism, the present study explores the association between obesity and LTHC with presenteeism using Generalized Estimating Equation (GEE) with a logistic link function. The econometric model developed to capture the association is as follows.

$$Y_{it}=\propto_{0}+\beta_{1}BMI_{it}+\beta_{2}LTHC_{it}+\beta_{3}SD_{it}+\beta_{4}LS_{it}+\beta_{5}JR_{it}+\varepsilon_{it}$$ (1)

In Eq 1, *Y_it_* represents presenteeism that a worker *i* may experience in period *t*; *BMI_it_* is the indicator of obesity, and *LTHC_it_* is the indicator of long term health condition. Finally, *SD_it_,LS_it_, and JR_it_* represent the vector of socio-demographic, lifestyle and job-related characteristics, respectively and *ε_it_* is the error term.

In the case of longitudinal data, repeated measurements on the same adult have been collected over time. For example, data on presenteeism, weight status, and LTHC of the same adult were taken repeatedly over the study period. As a result, observations from an individual are correlated and failure to take into account this correlation may lead to bias estimates. GEE can take into account the correlation of within-individual data. GEE estimate is a quasi-likelihood method where first mean and covariance are important. In the case of longitudinal data, observations on each individual are correlated. As a result, the Generalized Linear Model (GLM) cannot estimate parameters and make inferences as it assumes errors are independent and distributed individually. GEE can handle this issue by relaxing the assumption that observations were generated from a certain distribution. GEE estimates the population-averaged effects of the parameters. The main advantage of using GEE is that it is computationally simpler compared with Maximum Likelihood Estimates (MLE) in the case of categorical data. Besides, GEE offers a better prediction of the within-subject covariance structure. The main limitation of the GEE estimate is that likelihood-based methods cannot be applied to estimate the statistical inference.

This study revealed the adjusted association between obesity and LTHC with presenteeism by incorporating socio-demographic (age, gender, civil status, education, ethnicity, remoteness, and equivalized household income), lifestyle (smoking status, alcohol consumption, and physical activity) and job-related characteristics (hours worked per week, employment contract, occupation, industry, supervisory responsibilities, member of an employee association, paid sick leave and overall job satisfaction). The study results are presented in the form of Odds Ratio (OR) for each explanatory variable. This study set a P-value at <0.05 level for statistical significance. All statistical analyses were conducted using Stata version 16, Windows version.

### Ethics approval

This study requires no ethics approval for the authors as the analysis used only de-identified existing unit record data from the HILDA survey. However, the authors completed and signed the Confidentiality Deed Poll and sent it to NCLD (ncldresearch@dss.gov.au) and ADA (ada@anu.edu.au) before the data applications’ approval. Therefore, datasets analyzed and/or generated during the current study are subject to the signed confidentiality deed.

## Results

Table 1 provides a summary of the prevalence of presenteeism, BMI class, presence of LTHC, socio-demographic, lifestyle and employment characteristics of the study participants. A total of 111,086 workers were included in the final analysis. Among the participants, approximately 19% of workers reported presenteeism. Table 1 showed that approximately 35% of workers were overweight, 22% were obese and 16% had LTHC.

**Table 1:** Background characteristics of the study participants.

Variables	n	% (95% CI)
**Outcome variable: Presenteeism**		
*No*	90,172	81.17 (80.94–81.40)
*Yes*	20,914	18.83 (18.60–19.06)
**Health-related characteristics**		
BMI categories		
*BMI (<25)*	47,723	42.96 (42.67–43.25)
*Overweight (25*.*00–29*.*99)*	38,564	34.72 (34.44–35.10)
*Obesity (≥30)*	24,799	22.32 (22.08–22.57)
Long term health condition		
*No*	92,955	83.68 (83.46–83.89)
*Yes*	18,131	16.32 (16.11–16.54)
**Socio-demographic characteristics**		
Age		
*15–35 years*	46,943	42.26 (41.97–42.55)
*36–55 years*	50,047	45.05 (44.76–45.34)
*56–64 years*	14,096	12.69 (12.49–12.89)
Gender		
*Male*	56,126	50.52 (50.23–50.82)
*Female*	54,960	49.48 (49.18–49.77)
Civil status		
*Married / partnered)*	69,914	62.94 (62.65–63.22)
*Non-cohabitating*	41,172	37.06 (36.78–37.35)
Education		
*Year 12 or below*	40,270	36.25 (35.97–36.53)
*Professional qualification*	37,150	33.44 (33.17–33.72)
*University qualification*	33,666	30.31 (30.04–30.58)
Ethnicity		
*Not of indigenous origin*	108,323	97.51 (97.42–97.60)
*ATSI*	2,763	2.49 (2.40–2.58)
Remoteness		
*Major Cities*	76,583	68.94 (68.67–69.21)
*Regional*	32,862	29.58 (29.31–29.85)
*Remote or very remote*	1,641	1.48 (1.41–1.55)
Household income quintile		
*Quintile 1 (bottom quintile)*	16,592	14.94 (14.73–15.15)
*Quintile 2*	20,722	18.65 (18.43–18.88)
*Quintile 3*	22,763	20.49 (20.25–20.73)
*Quintile 4*	25,289	22.77 (22.52–23.01)
*Quintile 5 (top quintile)*	25,720	23.15 (22.91–23.40)
**Lifestyle factors**		
Smoking status		
*Non-smoker*	89,749	80.79 (80.56–81.02)
*Current Smoker*	21,337	19.21 (18.98–19.44)
Alcohol consumption		
*Former/non-drinker*	14,279	12.85 (12.66–13.05)
*Current drinker*	96,807	87.15 (86.95–87.34)
Physical activity		
*Inactive*	29,499	26.56 (26.30–26.82)
*Some activity*	35,845	32.27 (31.99–32.54)
*Regular activity*	45,742	41.18 (40.89–41.47)
**Job-related characteristics**		
Farm Size		
*Small*	47,902	43.12 (42.83–43.41)
*Medium*	30,658	27.60 (27.34–27.86)
*Large*	32,526	29.28 (29.01–29.55)
Hours worked/week		
*<35 hours a week*	36,153	32.55 (32.27–32.82)
*35–40 hours a week*	40,110	36.11 (35.83–36.39)
*>40 hours a week*	34,823	31.35 (31.08–31.62)
Employment contract		
*Permanent*	74,694	67.24 (66.96–67.52)
*Casual*	10,836	9.75 (9.58–9.93)
*Fixed-term*	25,556	23.01 (22.76–23.25)
Occupation		
*Professional*	27,209	24.49 (24.24–24.75)
*Managerial*	14,550	13.10 (12.90–13.30)
*Technical trade*	14,596	13.14 (12.94–13.34)
*Personal services*	12,809	11.53 (11.34–11.72)
*Clerical*	15,878	14.29 (14.09–14.50)
*Sales*	10,007	9.01 (8.84–9.18)
*Machinery*	6,373	5.74 (5.60–5.88)
*Labour work*	9,664	8.70 (8.54–8.87)
Industry		
*Public services*	7,444	6.70 (6.56–6.85)
*Agriculture*	2,681	2.41 (2.32–2.51)
*Mining*	1,972	1.78 (1.70–1.85)
*Manufacturing*	8,911	8.02 (7.86–8.18)
*Electricity*	1,104	0.99 (0.93–1.05)
*Construction*	8,938	8.05 (7.89–8.21)
*Trade*	14,621	13.16 (12.96–13.36)
*Hospitality*	7,153	6.44 (6.30–6.59)
*Transport*	6,943	6.25 (6.11–6.39)
*Finance*	4,006	3.61 (3.50–3.72)
*Education*	11,417	10.28 (10.10–10.42)
*Health*	15,819	14.24 (14.04–14.45)
*Other services*	20,077	18.07 (17.85–18.30)
Supervisory responsibilities		
*Yes*	50,524	45.48 (45.19–45.77)
*No*	60,562	54.52 (54.23–54.81)
Employee association		
*Yes*	26,021	23.42 (23.18–23.67)
*No*	85,065	76.58 (76.33–76.82)
Paid sick leave		
*Yes*	81,543	73.41 (73.14–73.66)
*No*	29,543	26.59 (26.34–26.86)
Overall job satisfaction (Mean [SD])	111,086	7.65 (1.62)

**Fig 2:**
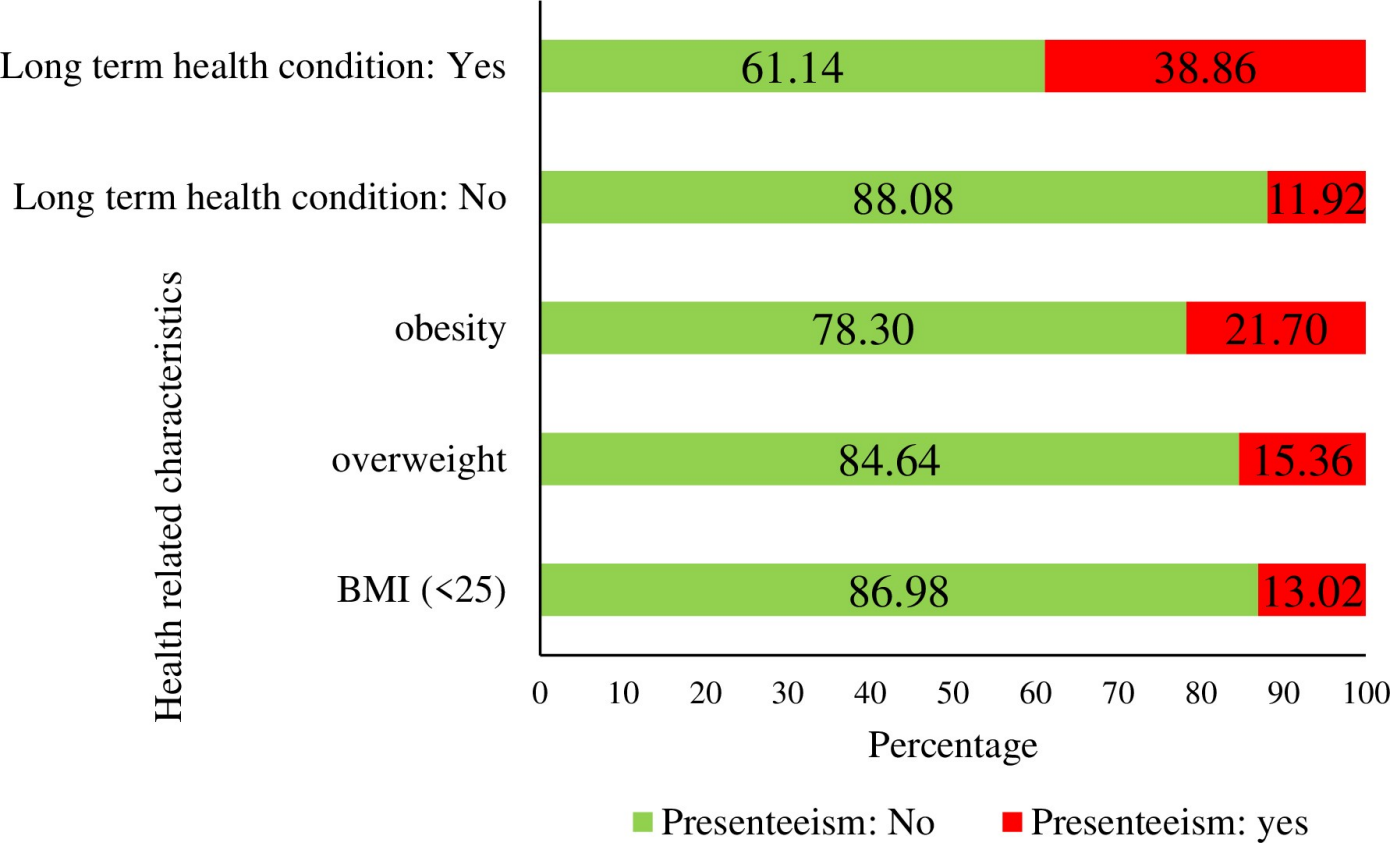
Prevalence of presenteeism by weight status and long term health condition.

Fig 2 demonstrates the reported presenteeism by weight status and presence of LTHC. There was a substantial difference in the prevalence of presenteeism by BMI categories and LTHC variables. The prevalence of presenteeism was found highest among obese workers (22%), following overweight (16%), and workers with BMI<25 (13%). Approximately, 39% of workers having LTHC reported presenteeism.

Table 2 presents the distribution of reported presenteeism by BMI categories, health, socio-demographic, lifestyle, and job-related characteristics. Table 2 also reports the bivariate relationship between presenteeism, obesity, LTHC along with other covariates achieved through the Chi-square tests or t-tests. The results showed that BMI, LTHC, and all the confounders were significantly associated with presenteeism in the bivariate analyses.

**Table 2:** Bivariate analysis between health, socio-demographic, lifestyle, and job-related characteristics with presenteeism in Australian workers.

Variables	No presenteeism		Presenteeism		P-value
	n	% (95% CI)	n	% (95% CI)	
**Health-related characteristics**					
BMI categories					<0.001
*BMI (<25)*	39,904	83.62 (83.28–83.95)	7,819	16.38 (16.05–16.72)	
*Overweight (25*.*00–29*.*99)*	31,708	82.22 (81.84–82.60)	6,856	17.78 (17.40–18.16)	
*Obesity (≥30)*	18,560	74.84 (74.30–75.38)	6,239	25.16 (24.62–25.70)	
Long term health condition					<0.001
*No*	80,047	86.11 (85.89–86.33)	12,908	13.89 (13.67–14.11)	
*Yes*	10,125	55.84 (55.12–56.57)	8,006	44.16 (43.43–44.88)	
**Socio-demographic characteristics**					
Age					<0.001
*15–35 years*	39,739	84.65 (84.32–84.98)	7,204	15.35 (15.02–15.68)	
*36–55 years*	39,952	79.83 (79.48–80.18)	10,095	20.17 (19.82–20.52)	
*56–64 years*	10,481	74.35 (73.63–75.07)	3,615	25.65 (24.93–26.37)	
Gender					<0.001
*Male*	46,766	83.32 (83.01–83.63)	9,360	16.68 (16.37–16.99)	
*Female*	43,406	78.98 (78.63–79.32)	11,554	21.02 (20.68–21.37)	
Civil status					<0.01
*Married / partnered)*	57,065	81.62 (81.33–81.91)	12,849	18.38 (18.09–18.67)	
*Non-cohabitating*	33,107	80.41 (80.03–80.79)	8,065	19.59 (19.21–19.97)	
Education					<0.01
*Year 12 or below*	32,986	81.91 (81.53–82.29)	7,284	18.09 (17.71–18.47)	
*Professional qualification*	29,814	80.25 (79.85–80.65)	7,336	19.75 (19.35–20.15)	
*University qualification*	27,372	81.30 (80.88–81.72)	6,294	18.70 (18.28–19.12)	
Ethnicity					<0.01
*Not of indigenous origin*	88,000	81.24 (81.00–81.47)	20,323	18.76 (18.53–19.00)	
*ATSI*	2,172	78.61 (77.04–80.10)	591	21.39 (19.90–22.96)	
Remoteness					<0.01
*Major Cities*	62,455	81.55 (81.28–81.83)	14,128	18.45 (18.17–18.72)	
*Regional*	26,349	80.18 (79.75–80.61)	6,513	19.82 (19.39–20.25)	
*Remote or very remote*	1,368	83.36 (81.48–85.09)	273	16.64 (14.91–18.52)	
Household income quintile					<0.001
*Quintile 1 (bottom quintile)*	13,017	78.45 (77.82–79.07)	3,575	21.55 (20.93–22.18)	
*Quintile 2*	16,620	80.20 (79.66–80.74)	4,102	19.80 (19.26–20.34)	
*Quintile 3*	18,304	80.41 (79.89–80.92)	4,459	19.59 (19.08–20.11)	
*Quintile 4*	20,799	82.25 (81.77–82.71)	4,490	17.75 (17.29–18.23)	
*Quintile 5 (top quintile)*	21,432	83.33 (82.87–83.78)	4,288	16.67 (16.22–17.13)	
**Lifestyle factors**					
Smoking status	73,379	81.76 (81.51–82.01)	16370	18.24 (17.99–18.49)	<0.001
*Non-smoker*	16,793	78.70 (78.15–79.25)	4,544	21.30 (20.75–21.85)	
*Current Smoker*					
Alcohol consumption					<0.001
*Former/non-drinker*	10,948	76.67 (75.97–77.36)	3,331	23.33 (22.64–24.03)	
*Current drinker*	79,224	81.84 (81.59–82.08)	17,583	18.16 (17.92–18.41)	
Physical activity					<0.001
*Inactive*	23,282	78.92 (78.46–79.39)	6,217	21.08 (20.61–21.54)	
*Some activity*	29,095	81.17 (80.76–81.57)	6,750	18.83 (18.43–19.24)	
*Regular activity*	37,795	82.63 (82.28–82.97)	7,947	17.37 (17.03–17.72)	
**Job-related characteristics**					
Farm Size					<0.001
*Small*	38,510	80.39 (80.04–80.75)	9,392	19.61 (19.25–19.96)	
*Medium*	25,148	82.03 (81.59–82.45)	5,510	17.97 (17.55–18.41)	
*Large*	26,514	81.52 (81.09–81.93)	6,012	18.48 (18.07–18.91)	
Hours worked/week					<0.001
*<35 hours a week*	28,288	78.25 (77.82–78.67)	7,865	21.75 (21.33–22.18)	
*35–40 hours a week*	33,049	82.40 (82.02–82.77)	7,061	17.60 (17.23–17.98)	
*>40 hours a week*	28,835	82.80 (82.40–83.20)	5,988	17.20 (16.80–17.60)	
Employment contract					<0.001
*Permanent*	61,060	81.75 (81.47–82.02)	13,634	18.25 (17.98–18.53)	
*Casual*	8,865	81.81 (81.07–82.53)	1,971	18.19 (17.47–18.93)	
*Fixed-term*	20,247	79.23 (78.72–79.72)	5,309	20.77 (20.28–21.28)	
Occupation					<0.001
*Professional*	22,060	81.08 (80.61–81.54)	5,149	18.92 (18.46–19.39)	
*Managerial*	11,986	82.38 (81.75–82.99)	2,564	17.62 (17.01–18.25)	
*Technical trade*	12,078	82.75 (82.13–83.35)	2,518	17.25 (16.65–17.87)	
*Personal services*	10,093	78.80 (78.08–79.50)	2,716	21.20 (20.50–21.92)	
*Clerical*	12,941	81.50 (80.89–82.10)	2,937	18.50 (17.90–19.11)	
*Sales*	8,199	81.93 (81.17–82.67)	1,808	18.07 (17.33–18.83)	
*Machinery*	5,193	81.48 (80.51–82.42)	1,180	18.52 (17.58–19.49)	
*Labour work*	7,622	78.87 (78.04–79.67)	2,042	21.13 (20.33–21.96)	
Industry					<0.001
*Public services*	6,076	81.62 (80.73–82.49)	1,368	18.38 (17.51–19.27)	
*Agriculture*	2,052	76.54 (74.90–78.10)	629	23.46 (21.90–25.10)	
*Mining*	1,667	84.53 (82.87–86.06)	305	15.47 (13.94–17.13)	
*Manufacturing*	7,327	82.22 (81.42–83.00)	1,584	17.78 (17.00–18.58)	
*Electricity*	927	83.97 (81.68–86.02)	177	16.03 (13.98–18.32)	
*Construction*	7,489	83.79 (83.01–84.54)	1,449	16.21 (15.46–16.99)	
*Trade*	12,034	82.31 (81.68–82.92)	2,587	17.69 (17.08–18.32)	
*Hospitality*	5,787	80.90 (79.98–81.80)	1,366	19.10 (18.20–20.02)	
*Transport*	5,633	81.13 (80.19–82.04)	1,310	18.87 (17.96–19.81)	
*Finance*	3,379	84.35 (83.19–85.44)	627	15.65 (14.56–16.81)	
*Education*	9,143	80.08 (79.34–80.80)	2,274	19.92 (19.20–20.66)	
*Health*	12,259	77.50 (76.84–78.14)	3,560	22.50 (21.86–23.16)	
*Other services*	16,399	81.68 (81.14–82.21)	3,678	18.32 (17.79–18.86)	
Supervisory responsibilities					<0.001
*Yes*	41,342	81.83 (81.49–82.16)	9,182	18.17 (17.84–18.51)	
*No*	48,830	80.63 (80.31–80.94)	11,732	19.37 (19.06–19.69)	
Employee association					<0.001
*Yes*	20,599	79.16 (78.67–79.65)	5,422	20.84 (20.35–21.33)	
*No*	69,573	81.79 (81.53–82.05)	15,492	18.21 (17.95–18.47)	
Paid sick leave					<0.001
*Yes*	66,664	81.75 (81.49–82.02)	14,879	18.25 (17.98–18.51)	
*No*	23,508	79.57 (79.11–80.03)	6,035	20.43 (19.97–20.89)	
Overall job satisfaction	90,172	7.73 (1.56)	20,914	7.32 (1.81)	<0.001

Table 3 displays the estimates of the association between obesity, LTHC, and presenteeism. To facilitate interpretation, this study presents the results in the form of odds ratios which indicate a change in the odds of presenteeism associated with a change in the level of an explanatory variable. The present study found that both obesity and LTHC were significant predictors of high presenteeism at work. The adjusted model demonstrates that the odds of presenteeism among the overweight and obese workers were 1.09 (95% CI: 1.05–1.14) and 1.38 (95% CI: 1.31–1.45) times higher, respectively, compared with workers with BMI<25. The results also revealed that workers having LTHC were 3.03 times (95% CI: 2.90–3.16) more likely to report presenteeism compared with peers not having LTHC.

**Table 3:** Multivariate analysis using Generalized Estimating Equation for factors associated with presenteeism^a^.

Variables	Fully adjusted model
	OR (95% CI), *P*-value
**Health-related characteristics**	
BMI categories	
*BMI (<25) (ref)*	
*Overweight (25*.*00–29*.*99)*	**1.09 (1.05–1.14), <0.001**
*Obesity (≥30)*	**1.38 (1.31–1.45), <0.001**
Long term health condition (LTHC)	
*No (ref)*	
*Yes*	**3.03 (2.90–3.16), <0.001**
**Socio-demographic characteristics**	
Age	
*15–35 years (ref)*	
*36–55 years*	**1.22 (1.16–1.27), <0.001**
*56–64 years*	**1.45 (1.36–1.55), <0.001**
Gender	
*Male (ref)*	
*Female*	**1.29 (1.23–1.36), <0.001**
Civil status	
*Married / partnered (ref)*	
*Non-cohabitating*	**1.07 (1.02–1.11), 0.005**
Education	
*Year 12 or below (ref)*	
*Professional qualification*	**1.10 (1.04–1.16), 0.001**
*University qualification*	**1.13 (1.05–1.20), <0.001**
Ethnicity	
*Not of indigenous origin (ref)*	
*ATSI*	1.11 (0.97–1.26), 0.119
Remoteness	
*Major Cities*	
*Regional*	1.01 (0.96–1.06), 0.795
*Remote or very remote*	0.92 (0.78–1.08), 0.317
Household income quintile	
*Quintile 1 (bottom quintile)*	**1.11 (1.05–1.18), <0.001**
*Quintile 2*	1.05 (0.99–1.10), 0.114
*Quintile 3*	1.00 (0.95–1.06), 0.994
*Quintile 4*	0.99 (0.94–1.04), 0.786
*Quintile 5 (top quintile) (ref)*	
**Lifestyle factors**	
Smoking status	
*Non-smoker (ref)*	
*Current Smoker*	** 1.20 (1.15–1.26), <0.001**
Alcohol consumption	
*Former/non-drinker (ref)*	
*Current drinker*	**0.75 (0.72–0.80), <0.001**
Physical activity	
*Inactive (ref)*	
*Some activity*	**0.68 (0.65–0.72), <0.001**
*Regular activity*	**0.53 (0.50–0.56), <0.001**
**Job-related characteristics**	
Farm Size	
*Small (ref)*	
*Medium*	**0.93 (0.89–0.98), 0.003**
*Large*	0.96 (0.91–1.00), 0.071
Hours worked/week	
*<35 hours a week*	**1.10 (1.05–1.15), <0.001**
*35–40 hours a week (ref)*	
*>40 hours a week*	0.97 (0.93–1.02), 0.202
Employment contract	
*Permanent (ref)*	
*Casual*	1.04 (0.97–1.13), 0.304
*Fixed-term*	0.97 (0.91–1.03), 0.283
Occupation	
*Professional (ref)*	
*Managerial*	0.97 (0.90–1.04), 0.343
*Technical trade*	1.03 (0.95–1.12), 0.431
*Personal services*	1.04 (0.97–1.12), 0.293
*Clerical*	0.93 (0.87–1.01), 0.052
*Sales*	1.00 (0.92–1.09), 0.978
*Machinery*	0.98 (0.88–1.08), 0.681
*Labour work*	1.08 (0.99–1.18), 0.075
Industry	
*Public services (ref)*	
*Agriculture*	1.15 (0.98–1.34), 0.083
*Mining*	1.01 (0.85–1.19), 0.942
*Manufacturing*	0.93 (0.83–1.03), 0.152
*Electricity*	0.92 (0.75–1.11), 0.367
*Construction*	0.98 (0.88–1.09), 0.701
*Trade*	0.91 (0.83–1.01), 0.075
*Hospitality*	0.94 (0.84–1.04), 0.236
*Transport*	0.99 (0.88–1.10), 0.795
*Finance*	**0.86 (0.75–0.98), 0.024**
*Education*	0.99 (0.89–1.09), 0.795
*Health*	1.00 (0.91–1.10), 0.992
*Other services*	0.96 (0.88–1.05), 0.429
Supervisory responsibilities	
*Yes (ref)*	
*No*	0.97 (0.94–1.01), 0.157
Employee association	
*Yes (ref)*	
*No*	**0.93 (0.89–0.98), 0.004**
Paid sick leave	
*Yes (ref)*	
*No*	0.98 (0.91–1.05), 0.537
Overall job satisfaction (from 0 = worst to 10 = best)	** 0.91 (0.90–0.92), <0.001**

Abbreviations: OR Odds Ratios; CI Confidence Interval; Ref Reference.

^a^Values in bold are statistically significant at p<0.05.

## Discussion

This population-based study found that the main effect of obesity and LTHC is strikingly similar. The study showed positive associations between obesity and LTHC with presenteeism among workers in different occupations in Australia.

Obese workers have higher odds of presenteeism than non-obese workers (BMI<25). The large disparity in the odds of diminished productivity at work associated with obesity is expected given that participants were explicitly asked about productivity loss stemming from physical problems. This finding is in line with previous studies where obesity has been identified as a strong predictor of presenteeism [10, 11]. Other observational studies conducted in the US have confirmed that obesity had a negative impact on work through presenteeism [9, 12, 13]. However, a recent study using a cross-sectional correlational design found that BMI was unrelated to presenteeism [23].

Presenteeism at work may occur due to health problems, such as the functional limitations of the workers. Another striking finding of the present study is that LTHC is linked to an increase in the odds of presenteeism. This finding is in line with an earlier study that found employees with chronic health conditions report higher rates of presenteeism compared with peers without having such health conditions [14]. A prior study also revealed that workers with moderate and severe functional limitations due to health problems were 1.28 and 1.63 times, respectively, more likely to report productivity loss at work [25]. Besides, a recent study claimed that the likelihood of presenteeism is higher among workers with chronic health conditions [10]. However, this finding is contrary to other studies that have suggested that health conditions, such as allergies, asthma, arthritis, back pain, sinus problems, broken bones, heart disease, cancer, and diabetes are not associated with presenteeism in the workplace [23].

There are several reasons behind the positive association between obesity and LTHC with work productivity impairment. Obese workers often face difficulty in moving due to bodyweight/size and excess adiposity. Moreover, body pain, musculoskeletal pain, osteoarthritis, and rheumatoid arthritis are often associated with weight gain [26]. The presence of these co-morbidities may limit obese workers’ ability to move without pain or discomfort and could result in productivity impairment in a physically demanding job [27]. Another possible explanation is that obese workers with sleep apnea and heart disease may experience weakness and dyspnea (shortness of breath). These health conditions make workers tired or slow to complete their job tasks on time [13].

The study findings confirm the need for effective interventions to reduce obesity in workers and improve their productivity at work. At present, the workplace has been considered as a potential avenue through which interventions could be implemented for managing healthy weight [28]. The findings of this study are expected to serve as useful evidence to health policymakers and employers to initiate workplace-based interventions to combat the obesity epidemic at work and thus reducing the productivity loss of the workers. Organizations should focus on multi-pronged interventions, such as providing information, social support for promoting a healthy lifestyle, and modification of the work environment to facilitate weight management of employees. For example, organizations may introduce sit-stand desks to reduce sitting time at work among desk-based workers, offer healthier food choices in cafeteria menus and vending machines, encourage walking during breaks, support active commuting options, provide educational modules on physical activity, diet, and lifestyle change, and establish gym and activity centers for performing physical activities.

The present study offers an important contribution to the existing body of knowledge by revealing a longitudinal association between obesity and LTHC with workplace performance by using data of 111,086 Australian workers from 2006 through 2018. In the existing literature, the majority of studies were cross-sectionally designed and thus cannot reveal the within-person change in presenteeism due to obesity and LTHC. The present study has several important strengths. First, is that it measured presenteeism using three comprehensive questions. Many of the previous studies assessed presenteeism through a single question [11, 29, 30] and it is difficult to establish the validity of presenteeism measure through a single question. Moreover, this study incorporated a large number of employment controls including less investigated variables (supervisory responsibilities, member of employee association or union, paid sick leave, and overall job satisfaction) to precisely estimate the association between obesity and LTHC with presenteeism. Additionally, this study fills the gap of the lack of studies on the longitudinal association between obesity and LTHC with presenteeism.

The present study has certain limitations that should be considered when interpreting the findings. First, the study results might be vulnerable to self-reported bias, as data on BMI and presenteeism along with other covariates were self-reported. Previous studies demonstrated that self-reported BMI is usually less than actual BMI as respondents tend to underreport weight and overreport height [31, 32]. Besides, this study’s unbalanced longitudinal research design prevents inferring the direction of causality. Given these limitations, the present study calls for prospective research that may capture the within-person change in presenteeism due to obesity and LTHC.

## Conclusion and recommendations

In summary, the present study utilized a large nationally representative dataset over the period from 2006 to 2018 to examine the longitudinal association between obesity, LTHC, and presenteeism. The study findings demonstrated that obesity and LTHC have longitudinal associations with presenteeism, independent of health, socio-demographic, lifestyle, and job-related confounders. Overweight and obesity among workers increases the costs of employers as overweight and obese workers reported higher presenteeism than under and normal-weight counterparts (BMI<25) at work. This study adds evidence to the existing literature that has shown the negative impact of obesity on presenteeism.

Presenteeism is a perennial and costly problem that should be tackled. The study findings stress the importance of health promotion, more specifically promoting healthy weight maintenance to reduce presenteeism or productivity loss at work. Maintaining healthy weight among workers through a healthy lifestyle may result in lower presenteeism, leading to socio-economic benefits for individual workers, employers, and society as a whole.

## Abbreviation

ATSI: Aboriginal or Torres Strait Islander
AUD: Australian Dollar
BMI: Body Mass Index
HILDA: Household, Income and Labour Dynamics in Australia survey
LTHC: Long Term Health Condition
OR: Odds Ratio
WHO: World Health Organization
